# PPAR-γ Agonists As Antineoplastic Agents in Cancers with Dysregulated IGF Axis

**DOI:** 10.3389/fendo.2017.00031

**Published:** 2017-02-22

**Authors:** Veronica Vella, Maria Luisa Nicolosi, Stefania Giuliano, Maria Bellomo, Antonino Belfiore, Roberta Malaguarnera

**Affiliations:** ^1^Scienze delle Attività Motorie e Sportive, University Kore, Enna, Italy; ^2^Endocrinology, Department of Health Sciences, University Magna Graecia of Catanzaro, Catanzaro, Italy

**Keywords:** insulin/IGF signaling pathway, insulin resistance, hyperinsulinemia, PPAR-γ, cancer, thiazolidinediones

## Abstract

It is now widely accepted that insulin resistance and compensatory hyperinsulinemia are associated to increased cancer incidence and mortality. Moreover, cancer development and progression as well as cancer resistance to traditional anticancer therapies are often linked to a deregulation/overactivation of the insulin-like growth factor (IGF) axis, which involves the autocrine/paracrine production of IGFs (IGF-I and IGF-II) and overexpression of their cognate receptors [IGF-I receptor, IGF-insulin receptor (IR), and IR]. Recently, new drugs targeting various IGF axis components have been developed. However, these drugs have several limitations including the occurrence of insulin resistance and compensatory hyperinsulinemia, which, in turn, may affect cancer cell growth and survival. Therefore, new therapeutic approaches are needed. In this regard, the pleiotropic effects of peroxisome proliferator activated receptor (PPAR)-γ agonists may have promising applications in cancer prevention and therapy. Indeed, activation of PPAR-γ by thiazolidinediones (TZDs) or other agonists may inhibit cell growth and proliferation by lowering circulating insulin and affecting key pathways of the Insulin/IGF axis, such as PI3K/mTOR, MAPK, and GSK3-β/Wnt/β-catenin cascades, which regulate cancer cell survival, cell reprogramming, and differentiation. In light of these evidences, TZDs and other PPAR-γ agonists may be exploited as potential preventive and therapeutic agents in tumors addicted to the activation of IGF axis or occurring in hyperinsulinemic patients. Unfortunately, clinical trials using PPAR-γ agonists as antineoplastic agents have reached conflicting results, possibly because they have not selected tumors with overactivated insulin/IGF-I axis or occurring in hyperinsulinemic patients. In conclusion, the use of PPAR-γ agonists in combined therapies of IGF-driven malignancies looks promising but requires future developments.

## Introduction

Peroxisome proliferator activated receptors (PPARs) are transcription factors that regulate gene expression and repression upon binding to natural or synthetic ligands (1). PPARs belong to the nuclear hormone receptor superfamily that includes receptors for steroids, thyroid hormones, vitamin D, and retinoic acid. Different subtypes of PPARs called PPAR-α, PPAR-β, PPAR-γ, and PPAR-δ have been identified. Each of them displays differential tissue distribution and mediates specific functions in early development, cell proliferation, differentiation, apoptosis, and metabolic homeostasis (1). PPAR-γ is expressed at high levels in adipose tissue and at lower levels in several other tissues, such as breast, colon, lung, ovary, prostate, and thyroid (1, 2). Many synthetic PPAR-γ ligands have been developed. The most widely used synthetic agents belong to the thiazolidinedione (TZD) class of antidiabetic drugs (also referred to as glitazones or TZDs) that includes ciglitazone, troglitazone, pioglitazone (PIO), and rosiglitazone (RGZ). Some glitazones are already in the clinical use as insulin sensitizers in patients with type 2 diabetes mellitus (T2DM) (3). Activation of PPAR-γ plays an inhibitory role in cell growth and proliferation by favoring cell differentiation (4). These properties make PPAR-γ activation by natural and synthetic ligands an attractive option in cancer prevention and treatment. However, PPAR-γ ligands exert their effects through both PPAR-γ dependent and independent pathways, often triggering cross talks with other signaling pathways, including the insulin-like growth factor (IGF) system signaling.

**Figure 1:**
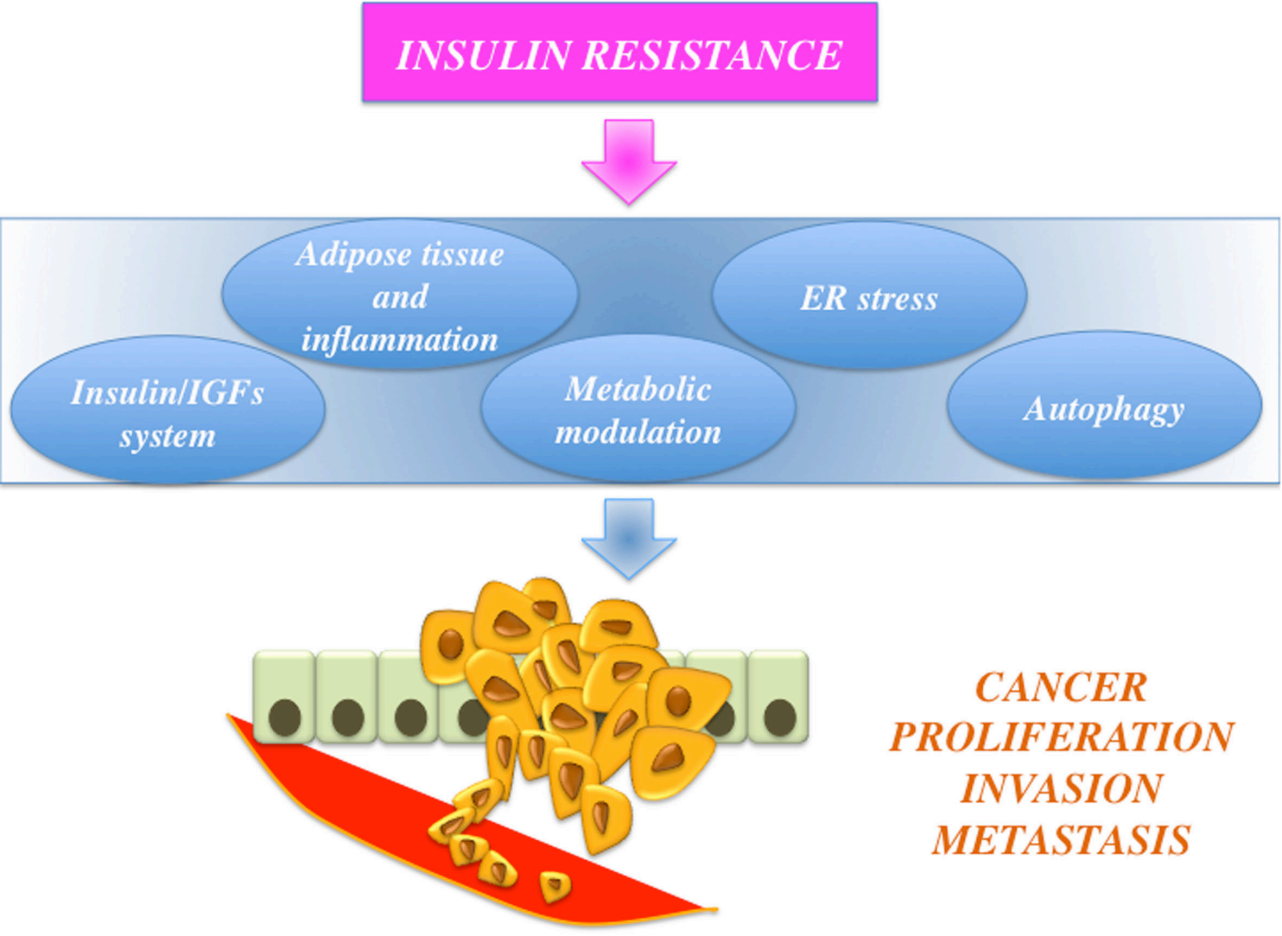
**Schematic representation of the links between obesity, insulin resistance, and cancer**. Insulin resistance is associated with protumoral actions, such as insulin-like growth factors (IGFs) axis hyperactivation, adiposity-related low-grade inflammation, and modulation of cell metabolism, endoplasmic reticulum (ER) stress, and altered autophagy.

Dysregulated activation of IGF axis has recently emerged as a relevant factor in development and progression of a variety of human malignancies (5–8). For instance, cancer cells are frequently characterized by altered expression of various components of the IGF including autocrine and/or paracrine secretion of IGFs (IGF-I and IGF-II) and overexpression of their cognate receptors [the IGF-I receptor, IGF-insulin receptor (IR), and the closely related IR]. In particular, IR overexpression may explain the increased sensitivity of cancer cells to hyperinsulinemia. Notably, in cancer cells, IR is often predominantly expressed as the “fetal” insulin receptor isoform A (IR-A), which binds both insulin and IGF-II (9). IR overexpression also contributes to enhanced signaling of IGF-II and IGF-I through the formation of IR/IGF-IR hybrid receptors (5). Not surprisingly, a number of epidemiological studies have consistently demonstrated that insulin resistance and hyperinsulinemia, common features of obesity and T2DM, are often associated with increased risk for several types of cancer (including cancers of the breast, colorectum, liver, and pancreas) (10–12) (Figure 1). One viable anticancer strategy is, therefore, to reduce insulin resistance and/or target the various IGF system components that are deregulated and that sustain the constitutive overactivation of IGF axis in cancer cells.

This review will focus on the mechanisms that link insulin resistance to cancer risk and will discuss the possible clinical implications of PPAR-γ agonists in cancers with dysregulated IGF axis.

## The Link Between Obesity, Insulin Resistance, and Cancer

Several epidemiological studies have consistently shown that metabolic disorders characterized by hyperinsulinemia and insulin resistance, such as obesity and T2DM, are associated with a significantly increased risk of cancer and cancer-specific mortality (13). The links underlying this association are not entirely clear and appear to involve a number of complex mechanisms.

### Mechanisms Involving the Insulin/IGFs System and Estrogens

Insulin is a major mediator of important metabolic functions. However, it is widely accepted that it may exert mitogenic functions through the activation of different signaling pathways. Notably, cancer cells usually overexpress molecules that are typical of fetal life, as they confer them a survival advantage compared to normal cells (14). Specifically, the IR-A, which is predominantly expressed by fetal and cancer cells, exerts proliferative and protumoral effects in response to insulin, proinsulin, and IGF-II. In contrast, the second isoform, IR-B, is mainly involved in metabolic functions (9, 15, 16). IR-A also binds IGF-I with a lower affinity than IGF-II. Both IGFs circulate in the bloodstream but they are also secreted at high levels by cancer cells and/or tumor stroma (17) thus activating IR-A in cancer cells (5, 18). Moreover, while in normoinsulinemic individuals the principal pathway activated by insulin to control whole-body metabolism is the PI3K cascade, in insulin resistant, hyperinsulinemic subjects this metabolic branch is blunted while the more mitogenic MAPK/mTOR cascade is overactivated. This unbalance between the MAPK and the PI3K cascades is associated with impaired glucose/lipid homeostasis in typical target tissues like liver, muscle, and adipose tissue and with increased cell proliferation in other tissues (12, 19). In brief, insulin resistance and hyperinsulinemia are considered the main determinants of cancer initiation/progression in diabetic/obese patients, and therefore the major preventable cancer risk factor in these subjects.

Moreover, hyperinsulinemia may increase the bioavailability of IGF-I and IGF-II through two mechanisms: (1) by inhibiting the synthesis of certain IGF-binding proteins, such as IGF-binding protein 1 (IGF-BP1) and IGF-binding protein 2 (IGF-BP2), (2) by increasing IGF-I hepatic production.

The increased bioavailability of IGFs may contribute to tumor progression through the stimulation of IGF-IR, IR/IGF-IR hybrids, and IR-A itself (8). Partner molecules can be further recruited by these activated receptors contributing to increase cancer proliferation and migration (20, 21). Finally, a cross talk between the insulin/IGF axis and estrogens has been extensively reported in tumors where both signaling pathways are involved in controlling cell survival and proliferation. The functional interactions between these signaling cascades may occur at multiple levels. For instance, increased circulating insulin reduces the sex hormone-binding globulin thus increasing bioavailable sex steroids (22), which are associated with a higher risk for estrogen-dependent cancers such as breast and endometrial cancers (23). Moreover, ligand-dependent and independent activation of estrogen receptors increases insulin/IGF-mediated growth effects in several tumors, such as neuroblastoma, pituitary adenomas, and cancers from breast and prostate (24–26). Additionally, in prostate cancer cells, activation of classical ERs (and of androgen receptors) located at the level of cell membrane induces IGF-IR upregulation *via* membrane-initiated steroid signaling and enhances IGF-mediated biological effects (27, 28). Yet, in breast cancer cells, ligand-activated IGF-IR and IR upregulate the non-classical estrogen receptor (GPER), which potentiates the protumoral actions of insulin/IGFs and estrogens (29). Overall, all these functional interactions between insulin/IGFs and estrogens may concur to cancer growth and progression (30, 31).

### Mechanisms Involving Adipose Tissue and Chronic Low-Grade Inflammation

A second mechanism by which obesity is associated with cancer (32) is related to the adipose tissue expansion. As individuals become obese and their adipocytes enlarge, adipose tissue undergoes molecular and cellular alterations affecting the local and systemic metabolism. Adipocyte function dysregulation and the associated chronic inflammation may also contribute to adiposity-induced tumorigenesis (33, 34) (Figure 1). Yet, the insufficient vascularization of the enlarged adipose tissue results in hypoxia, and infiltration by macrophages, T cells, and natural killer cells. These cells generate large amounts of pro-inflammatory cytokines, including tumor necrosis factor α and interleukin-6, which act as paracrine signaling molecules. Each of these factors might play an etiologic role in regulating malignant transformation and/or cancer progression. Moreover, adipose tissue within the tumor microenvironment actively contributes to tumor growth and metastasis by secreting leptin, adiponectin, free fatty acid (FFA), pro-angiogenic factors, and extracellular matrix constituents (35). Indeed, cancer-associated adipocytes (CAAs), in concert with cancer-associated fibroblasts and tumor-associated macrophages, may influence cancer cell survival (35).

### Mechanisms Involving Modulation of Tumor Metabolism

Emerging data suggest that obesity may stimulate cancer progression by affecting tumor metabolism (36–38). This mechanism has been particularly studied in tumors arising in close proximity to adipose tissue (39). Indeed, Nieman et al. demonstrated that adipocytes act as mediators of ovarian cancer metastases providing fatty acids to the cancer cells (39). This mechanism is not limited to ovarian cancer but can be extended to other cancers. According to a recent view, tumor cells grow in a complex microenvironment characterized by a dynamic exchange of metabolites between stromal cells (fibroblasts and adipocytes) and epithelial cancer cells. Stromal cells provide metabolites (lactate, ketones, glutamines, and fatty acids) that are used by cancer cells to generate energy by oxidative phosphorylation and β-oxidation (reverse Warburg effect) (40). Adipocytes localized in proximity to cancer cells undergo dedifferentiation into pre-adipocytes, and some of them are reprogrammed into CAAs. Fatty acids derived from lipolysis are released by CAAs and utilized by cancer cells to obtain energy from mitochondrial β-oxidation. This availability of energetic substrates in the tumor microenvironment promotes uncontrolled cancer cell growth and tumor progression (35) (Figure 1). Lipid metabolism is, therefore, a new target for the treatment of cancers where adipocytes are a major component of the microenvironment.

### Mechanisms Involving Endoplasmic Reticulum (ER) Stress

In obesity, ER stress is due to the increased protein synthesis caused by nutrient excess and elevated levels of saturated FFA.

Cancer cells have developed a capacity to survive under these extreme conditions through the modulation of the unfolded protein response (UPR) pathway. The components of the UPR pathway have also been implicated in cancer (41) and appear to be affected by glucose homeostasis (42, 43). In fact, in many cancers, glucose-regulated protein 78 (GPR78, an ER protein chaperone involved in adaptive response to ER stress) is overexpressed and correlates with cancer recurrence, therapeutic resistance, and stemness phenotype (44–48). Glucose and leptin can induce expression of GPR78 (49, 50), suggesting a link between diabetes and ER stress-related cancer features (Figure 1).

### Mechanisms Involving Autophagy

Autophagy is a natural and regulated, destructive process that is activated upon starvation in order to disassemble, unnecessary or dysfunctional cellular components (51) that are broken down and recycled through lysosomes (52). This process is regulated in a biphasic way through short- and long-term responses. Posttranslational protein modifications and protein–protein interactions mediate the rapid response, while nuclear transcriptional mechanisms are induced after a sustained stimulus (53). Autophagy was initially considered a survival strategy during starvation, but it has been recently demonstrated that it can lead to apoptosis if prolonged (autophagic cell death) (54, 55). Autophagy has opposite roles in tumorigenesis and tumor progression. Decreased baseline levels of autophagy are observed in many cancer cells compared to non-cancerous cells from the same tissue. Indeed, inhibition of autophagy can promote carcinogenesis by decreasing protein degradation thus increasing unrepaired and accumulated mutations (Figure 1). On the contrary, in response to hypoxia, acidosis, or nutrient deprivation, autophagic process is enhanced in cancer cells in the later stages of tumor progression. The cells in the inner part of the tumor increase autophagy in order to survive to the low nutrient and hypoxic microenvironment (56). For this reason, in the last years many cancer therapies have been aimed to modulate autophagy.

Multiple signaling pathways, including mTOR, AMPK, B-cell lymphoma 2 (Bcl-2)/Beclin 1 complex, and p53, play important roles in regulating autophagy (57). Insulin inhibits autophagy in several ways: first by activating mTOR in synergy with amino acids (58), which results in the phosphorylation and inhibition of unc-51-like autophagy activating kinase 1 (59); second by inducing Akt-mediated phosphorylation and inhibition of the transcription factor FoxO3, which controls the transcription of autophagy-related genes, including LC3 and Bnip3 (60); third by inhibiting the expression of autophagy-related genes, such as VPS34 and Atg12 in a FoxO1-dependent manner (61).

During insulin resistance, various dysfunctional/damaged components are retained leading to cellular stresses and/or inflammation. At the same time, hyperinsulinemia may inhibit autophagy (60) and favor tumorigenesis and tumor progression. In insulin-resistant mice, autophagy is suppressed in many tissues (61).

## PPAR-γ Agonists: History, Mechanisms of Action, and Clinical Use

### History

Peroxisome proliferator activated receptor-γ is a nuclear hormone receptor that is activated by multiple agonists. The name derives from the first identified member that belongs to a group of hepatocarcinogens that upregulate the proliferation of peroxisomes (62). The γ variant was first cloned from a *Xenopus* cDNA library, along with the α and β variants (63). In the same study, all three receptors were observed to have a role in the regulation of β-oxidation. PPARs perform many activities, mainly *via* endogenous ligands generated from fatty acids. For this reason, they are called lipid sensors. PPAR agonists have different affinities for PPARs, various pharmacokinetic profiles, and specific gene expression profiles (64, 65). Shortly after the role of PPAR-γ in adipocyte differentiation was characterized, potent synthetic ligands of PPAR-γ (TZDs) were discovered (66) and subsequently used in the therapy of T2DM patients to improve insulin sensitivity (67).

### Mechanisms of Action

**Figure 2:**
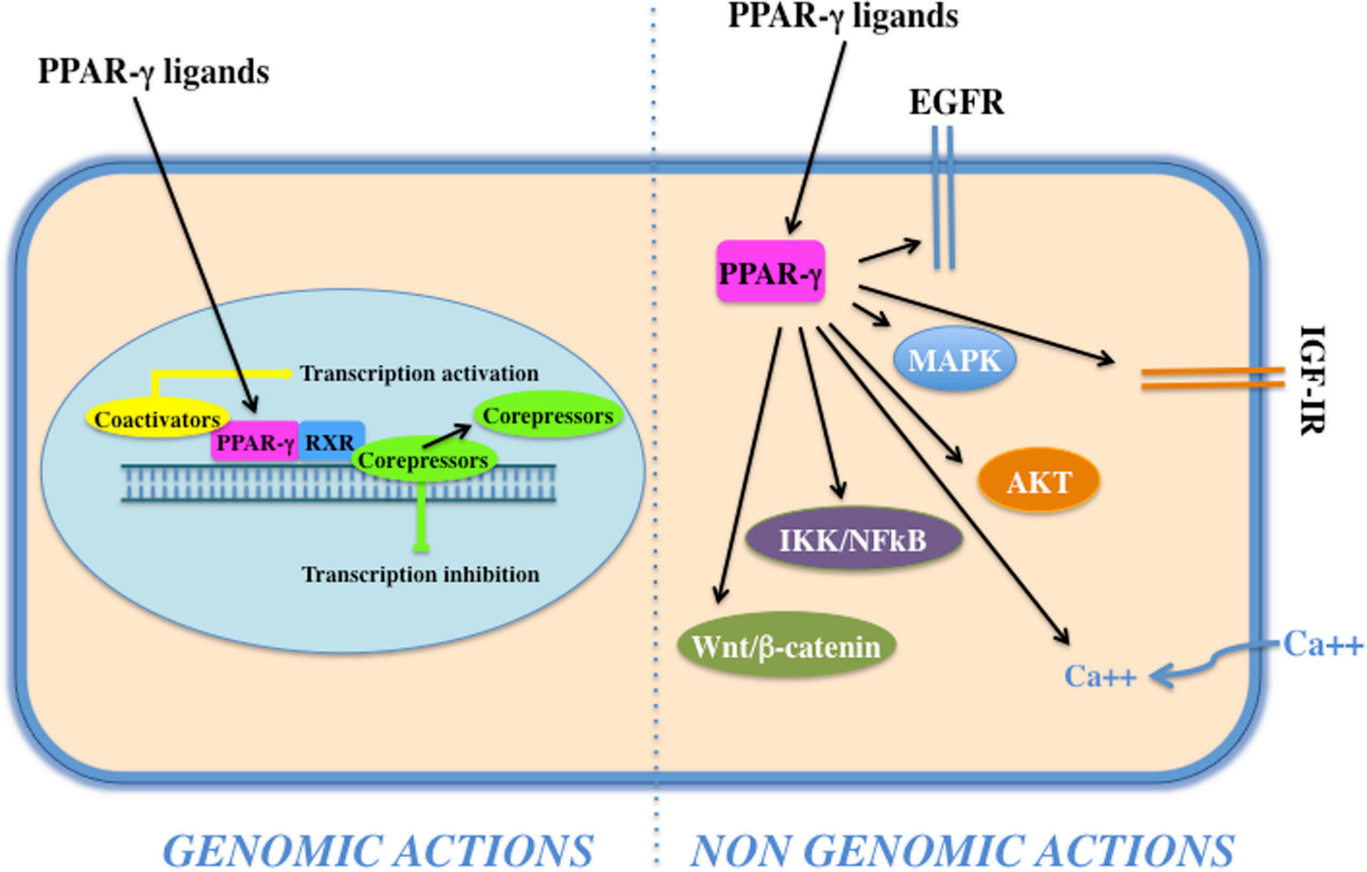
**Genomic and non-genomics actions of peroxisome proliferator activated receptor (PPAR)-γ**. PPAR-γ belongs to the class of nuclear receptors, containing a transactivation domain and a DNA-binding domain. Upon ligand binding, at the nuclear level, a conformational change leads to the release of corepressors, recruitment of coactivators, heterodimerization, and transactivation of PPAR-responsive element-related promoters (genomic actions). Cytoplasmic PPAR-γ, by interacting with proteins and activating transmembrane proteinases, elicits rapid and transient non-genomic effects that modulate EGF-R/insulin-like growth factor (IGF)-insulin receptor (IR) transactivation, calcium influx, PI3K/Akt, IKK/NFKB, and MAPKs signaling pathways.

Peroxisome proliferator activated receptor-γ forms a heterodimer with retinoid X receptor (RXR) and then binds to specific DNA sequences, named PPAR-responsive elements, in the promoter region of target genes. Binding of agonist ligands to the PPAR:RXR heterodimer activates the complex and initiates gene transcription. Alternatively, PPAR-γ can recruit transcriptional corepressor proteins to silence gene expression. PPAR-γ is activated by natural ligands such as polyunsaturated fatty acids, eicosanoids, oxidized low-density lipoproteins, J2 type prostaglandins, and by a panel of potent drug agonists that include the TZDs (68). Although PPAR-γ acts mainly as transcription factor (genomic action), it may also exert its function *via* the activation of cytosolic non-genomic signaling pathways (Figure 2). The latter comprises activation of transmembrane proteinases, EGF-R transactivation, calcium influx, changes in protein biosynthesis, modulation of mitochondrial functions, stress response, interaction with Wnt/β-catenin, induction of signaling pathways involved in proliferation and survival, such as IGF-I/PI3K/AKT/mTOR and MAPK (69). The cross talk with these cascades plays an important role in the regulation and signal transmission of PPAR-γ and its ligands (see Role of PPAR-γ Agonists in Cell Proliferation and Growth: In Vitro Evidences). Overall, the PPAR-γ non-genomic actions, the PPAR-γ genomic functions, and the modulation of the cross talk between PPAR-γ and other key survival pathways support the pleiotropic functions of PPAR-γ that include fat cell formation and differentiation (4, 70–73), glucose and lipid homeostasis (74–77), atherosclerosis regulation (78), and anti-inflammatory effects (79, 80). In addition, PPAR-γ and its ligands exert antiproliferative and anti-tumorigenic functions or induce pro-tumorigenic and antiapoptotic responses on the basis of the cell context (69).

### Clinical Use

Thiazolidinediones are potent insulin sensitizers that efficiently and sustainably improve glycemic control in patients with T2DM (81). Although both metformin and TZDs decrease hepatic glucose production (82, 83), only TZDs reduce liver fat content (82, 84). They also diminish fasting FFA concentrations (85). This effect probably accounts for the indirect improvement in skeletal-muscle insulin sensitivity and the reduction in liver steatosis.

However, the use of TZDs in clinical practice is currently limited because of side effects, such as weight gain and fluid retention that can precipitate cardiac failure and bone fractures. Troglitazone and RGZ were withdrawn because of hepatotoxicity (86) and suspected to increase cardiovascular risk (87), respectively. In addition, the benefit–risk ratio of PIO has been reassessed recently in light of a putatively increased risk of bladder cancer (see Role of PPAR-γ Agonists in Chemoprevention). A novel synthetic third-generation TZD highly selective for PPAR-γ, i.e., efatutazone (RS5444 and CS-7017) has been recently synthesized. So far, efatutazone is the most potent TZDs in terms of transcriptional response and cell proliferation inhibition (88).

## PPAR-γ Agonists as Antitumor Drugs: Cross Talk with the IGF System

**Figure 3:**
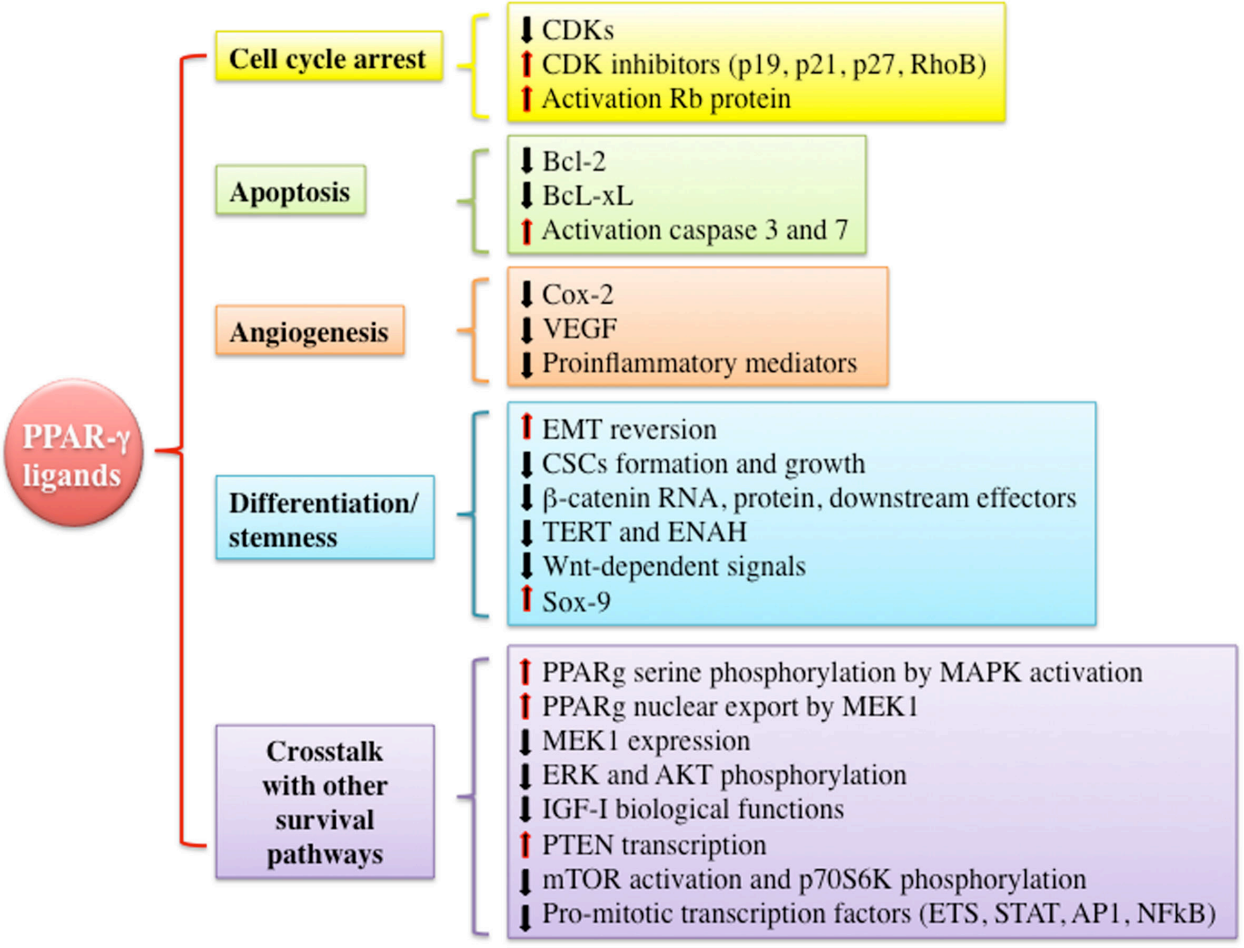
**Mechanisms underlying anti-tumorigenic actions of peroxisome proliferator activated receptor (PPAR)-γ agonists**. PPAR-γ activation by thiazolidinediones or other ligands may trigger anti-tumorigenic effects through different mechanisms including cell cycle arrest, induction of apoptosis, inhibition of angiogenesis, modulation of differentiation and stemness processes, and cross talk with other signaling pathways involved in proliferation and survival. All of these events may be exerted by PPAR-γ genomic or non-genomic actions. Abbreviations: CDKs, cyclin-dependent kinases; RhoB, rho-related GTP-binding protein; Rb protein, retinoblastoma-associated protein; Bcl-2, B-cell lymphoma 2; Bcl-xL, B-cell lymphoma-extra large; Cox-2, cyclooxygenase-2; VEGF, vascular endothelial growth factor; EMT, epithelial to mesenchymal transition; CSCs, cancer stem cells; TERT, telomerase reverse transcriptase; ENAH, enabled homolog; ETS, E26-transformation specific family; STAT, signal transducer and activator of transcription; AP1, activator protein-1.

As mentioned earlier, PPAR-γ and its ligands may exert pleiotropic effects (pro- vs. antineoplastic functions). However, PPAR-γ is often considered a tumor suppressor by the virtue of promoting growth inhibition, apoptosis, cell cycle arrest, and redifferentiation in several malignancies (Figure 3). *In vitro* and *in vivo* evidences suggest that many of these antineoplastic functions are explained by the interference with the IGF system activity at various levels and by the well-established metabolic actions, i.e., reduction of circulating insulin levels and improvement of tissue insulin sensitivity. These findings provide new insights for the potential clinical use of PPAR-γ agonists as anticancer agents especially in tumors characterized by IGFs’ overactivation and/or occurring in patients with insulin resistance and hyperinsulinemia.

### Role of PPAR-γ Agonists in Cell Proliferation and Growth: *In Vitro* Evidences

*In vitro* studies conducted in cancer cell lines derived from many tumors including breast cancer (89), lung cancer (90), colon (91), liposarcoma (92), hematopoietic cancer (93), glioma (94), hepatocarcinoma (95, 96), pancreatic cancer (97), thyroid cancer (98), and prostate cancer (99) have shown that PPAR-γ is often expressed in these cancer cells and that PPAR-γ activation exerts a growth inhibitory effect. Well-known molecular mechanisms implicated in the suppression of tumor growth and progression after PPAR-γ activation by TZDs or other PPAR-γ agonists involve cell cycle arrest, apoptosis, inhibition of angiogenesis, and redifferentiation. For instance, in cancer cells from different organs and tissues including the thyroid gland (98, 100, 101), lung (102, 103), esophagus (104), prostate (105), breast (106), kidney, and urothelium (107, 108), TZDs decrease cyclin-dependent kinase levels (CDKs) such as Cdk4, increase CDKs inhibitors such as p19, p21, p27, and rho-related GTP-binding protein, and activate retinoblastoma-associated protein. In some tumors, TZDs modulate proteins involved in apoptosis process. For example, in lung cancer cells, TGZ reduces the antiapoptotic protein Bcl-2; in anaplastic thyroid cancer, RGZ-induced apoptosis is associated with decrease of B-cell lymphoma-extra large expression and caspase-3 and caspase-7 activation; in many hepatoma cell lines, RGZ induces apoptosis by promoting the expression of PTEN, caspase-3, and caspase-9 (95, 96). In addition to apoptosis, PPAR-γ activation inhibits angiogenesis-associated proteins expression such as cyclooxygenase-2 and vascular endothelial growth factor (VEGF) as well as tumor microenvironment inflammatory mediators (79, 80, 97). Another mechanism by which PPAR-γ activation may act as tumor suppressor is the promotion of cellular differentiation (see next paragraph). Many of these PPAR-γ-mediated anticancer effects may be linked to a direct or indirect cross talk with the IGF axis. In particular, *in vitro* evidences indicate that PPAR-γ cooperates with the IGF axis downstream signaling pathways such as MAPK, PI3K, and mTOR. These interactions may occur in a synergistic or antagonistic way and in a context-specific manner, supporting the multifacet features of PPAR-γ effects. In accordance with the antitumor role of PPAR-γ and the antiapoptotic role of the IGF system and its downstream signaling pathways, MAPK activation by different growth stimuli (i.e., insulin, IGF-I, EGF, and PDGF) may induce PPAR-γ serine phosphorylation and consequently inactivation of its genomic activity (109–112). Indeed, poorly differentiated cancers typically show constitutive activation of mitogenic pathways downstream tyrosine-kinase receptors that are involved in chemoresistance (113). Yet, MEK1 interacting with PPAR-γ induces its nuclear export and inhibition of its genomic functions (114). On the other hand, PPAR-γ activation reduces MEK1 protein expression and ERK phosphorylation, causing cell growth arrest and apoptosis. Beyond the inhibition of MAPK pathway, PPAR-γ agonists may also inhibit other cascades downstream the insulin/IGF signaling, such as the PI3K/mTOR pathway. In particular, in several cancer cells from thyroid (98), liver (95, 111), colon (115), breast (115), lung (116, 117), and pancreas (118), PPAR-γ agonists inhibit IGF-I mediated biological effects through the reduction in Akt phosphorylation, increased PTEN expression levels (115), and inhibition of mTOR and p70S6K activity (116–120). Taken together, these data support the possibility that PPAR-γ agonists may have a potential role as antineoplastic agents in tumors characterized by overactivation of the insulin/IGF-I axis (121, 122).

At variance with these data, in some tumor cell models, a positive cross talk between PPAR-γ and MAPK/PI3K/mTOR signaling components may also occur. For example, in breast and colon cancer cells, TZDs have been described to induce rapid MAPK and PI3K/mTOR activation that may affect the antitumor PPAR-γ genomic action (115, 123, 124). These latter mechanisms add complexity to the actions of PPAR-γ agonists and may contribute to the absence of therapeutic efficacy of TZDs in some cancer patients.

### PPAR-γ Agonists and Autophagy

Peroxisome proliferator activated receptor-γ activation may revert the effect of hyperinsulinemia by favoring the autophagic process at multiple levels. For example, in breast cancer cells, PPAR-γ agonists induced autophagy through HIF-1α and BNIP3 upregulation (125), while in adrenocortical cancer cells, RGZ triggered autophagy by increasing the expression of AMPKα and beclin 1, through both PPAR-γ-dependent and PPAR-γ-independent mechanisms (126). In bladder cancer cells, TGZ treatment enhanced autophagy, and then apoptosis (127). Moreover, RGZ-induced autophagy depends on the cellular context since this effect was not observed in all cell lines. In some *in vitro* models, PPAR-γ ligands induce autophagy by increased ROS production in mitochondria thus altering the mitochondrial membrane potential (126).

### Role of PPAR-γ Agonists in Cell Differentiation and Stemness: *In Vitro* Evidences

As previously mentioned, PPAR-γ agonists may favor cancer cell differentiation (128). This notion is supported by experiments conducted in lung, breast, and thyroid cancer cells showing a change in epithelial expression profile and a reversion of epithelial–mesenchymal transition (EMT) process after TZD treatment (4, 98, 101, 111). Furthermore, several studies have demonstrated the efficacy of PPAR-γ agonists in inhibiting the survival of cancer stem cells (CSCs) derived from human cell lines or specimens from breast, prostate, colon, bladder, and blood tissues (129–134) supporting a role of PPAR-γ in regulating CSC biology. For example, PIO, in combination with a RXR ligand, was able to reduce the formation of mammospheres from human breast tumors and MCF7 cells (132). Yet, in bladder cancer cells, the combination of a natural ligand of PPAR-γ (i.e., 15d-PGJ2) together with a survivin inhibitor was associated with downregulation of stemness-related genes and reduction of spheres formation (134). The molecular mechanisms through which TZDs regulate differentiation and stemness programs have been studied in adipocytes and normal cells, while in cancer cells and in CSC they remain still incompletely elucidated. In adipocytes, PPAR-γ amplifies differentiation signals and inhibits proliferation by affecting the Wnt/GSK3-β/β-catenin pathway. In particular, PPAR-γ interacts with GSK3-β inducing the differentiation factor C/EBPα and leading to the production of adiponectin (70, 71); yet, PPAR-γ activation reduces β-catenin at both mRNA and protein levels promoting differentiation (135). Similar mechanisms involving the Wnt/β-catenin cascade may also occur in cancer cells and in CSC, as this pathway has also emerged to be essential not only for the differentiation process but also for self-renewal and stemness programs. In human kidney embryonic HEK293 cells and in human metastatic prostate cancer LnCaP cells, PPAR-γ suppresses Wnt signaling by targeting phosphorylated β-catenin to proteasome (136–138). In gastric and colon cancer cells, PPAR-γ inhibits β-catenin expression, subcellular localization, and downstream effectors. All of these events lead to the modulation of a subset of genes, such as telomerase reverse transcriptase, enabled homolog, and Sox9, involved in cell development, differentiation, and survival processes (139–141). Another mechanism through which PPAR-γ could modulate CSC biology is the cross talk with IGF signaling. Indeed, recent studies have provided increasing evidence that the IGF pathway is essential for the growth/expansion of cancer stem-like cells by contributing to regulate pluripotency, EMT, and self-renewal. We have recently found that IR and IGF-IR are overexpressed in human thyroid progenitor/stem cells where they regulate self-renewal ability and stem cell expansion (142–144). Similar findings have been demonstrated in cancer progenitor/stem cells from solid and hematopoietic cancers reinforcing the important role of the IGF system in regulating stem cell biology and the early steps of the carcinogenesis process (145, 146). Furthermore, to control cell reprogramming, the IGF system, in turn, interacts with effectors present in the stem cell niche belonging to GSK3-β/Wnt/β-catenin, Notch, and Shh pathways.

In this paragraph, we will focus only on Wnt/β-catenin signaling cascade, because there are more data regarding possible interactions between this pathway and both the IGF- and PPAR-γ-dependent signals. In particular, in human colon and hepatocellular cancer cells (147–149), IGF-I stimulates tyrosine phosphorylation of β-catenin, IRS-1, and E-cadherin as well as cellular relocation and stabilization of β-catenin. These events result in the disruption of β-catenin/E-cadherin interaction and inhibition of GSK3-β activity (148, 149). Furthermore, IRS-1, in turn, contributes to β-catenin stability, GSK3-β inactivation, IGF-IR/PI3K signaling pathway amplification, and T cell factor/lymphoid enhancer-binding factor-dependent transcription of genes controlling stem cell fate, long-term renewal, and differentiation programs (147). On the other hand, IRS-1 is a downstream target of β-catenin, which regulates IRS-1 expression and localization controlling cancer initiation, self-renewal, and differentiation processes (150–152). Overall, these interactions between GSK3-β/Wnt/β-catenin and the IGF system may contribute to enhance mitogenesis and stemness characteristics. In light of these considerations, PPAR-γ agonists, by inhibiting the activation of IGF axis as well as the GSK3-β/Wnt/β-catenin pathway, could be used in combination with other drugs such as inhibitors of tyrosine kinases (133), PI3K/AKT (153), and MAPK cascades to reach the maximum antitumor and pro-differentiating effect.

### Role of PPAR-γ Agonists As Antitumor Drugs and in Chemoprevention: Animal Models

Several *in vivo* studies conducted in mice and rats support the anticancer PPAR-γ properties (89, 99, 105, 154–159). In general, TZDs have shown univocal effects in impairing progression and metastatic spread in animals injected with human cancer cells. Less clear-cut results have been obtained when looking at the effect of PPAR-γ agonists in cancer prevention. The discrepancy between the anticancer and the tumor-promoting effects of PPAR-γ agonists shown in the different studies reflects, as seen *in vitro*, the complexity of signaling interactions involved in tumor formation *in vivo*. In some of these studies, the molecular mechanisms responsible for the antitumor vs. protumour PPAR-γ actions have been identified in the regulation of cell cycle, apoptosis, and signals involved in differentiation and cell fate such as the Wnt/β-catenin cascade and the Notch/Hes1 and NFKB pathways (105, 157, 159). However, so far, no studies in animals have explored the interactions between PPAR-γ and the IGF system or the correlation between PPAR-γ actions and the presence of insulin resistance in animals.

### PPAR-γ Agonists As Antitumor Drugs: Clinical Trials

Taken together, data from *in vitro* studies and from animal models strongly support the concept that PPAR-γ has antineoplastic effects. However, clinical trials, using TZDs as antineoplastic agents, are few and have reached conflicting results. Overall, the ambivalent findings in clinical trials may be due to the inclusion of pretreated refractory cancers or cancers in far advanced stages, or to the activation of PPAR-γ independent pathways.

Furthermore, most of these studies lack clinical information regarding the presence of insulin resistance (diabetes mellitus condition was a criterion of exclusion) as well as data regarding the alterations of IGF axis in the tumors studied. Few studies using PPAR-γ agonists and in combination with other drugs have been conducted. Encouraging results have been obtained in patients affected by chronic myeloid leukemia treated with TZDs and imatinib. Leukemia quiescent cells have been eliminated through a mechanism involving TZD-related inhibition of STAT5 expression responsible of stemness maintenance (129–131, 133). One relevant ongoing clinical phase II trial using PIO (15 mg) as add-on therapy to imatinib has started in July 2009 in patients affected by chronic myelogenous leukemia with residual molecular disease after imatinib monotherapy for more than 2 years (ACTIM EudraCT 2009-011675-79). Although the interim results from this trial are promising, the study was non-randomized. Yet, in hepatocellular carcinoma (HCC) *in vitro* and *in vivo* models, the use of RGZ in combination with either AKT pharmacological inhibitors or AKT siRNA significantly enhanced PPAR-γ agonist-mediated inhibition of cell proliferation, stem cell-like properties, and tumor growth (153). Although promising, further investigations regarding the antitumor effects of PPAR-γ agonists in the clinical setting need to be conducted. More data have been collected in diabetic populations with regard to the role of PPAR-γ agonists in chemoprevention (see next paragraph).

### Role of PPAR-γ Agonists in Chemoprevention: Lessons from Diabetic Patients Treated with TZDs

Thiazolidinediones are widely used as antidiabetic agents in T2DM patients. According to *in vivo* studies in diabetic hyperinsulinemic mice showing that TGZ has potent glucose and insulin-reducing effects (160), several reports have demonstrated that both TGZ and PIO reduce hyperglycemia, hyperinsulinemia, and hypertriglyceridemia and improve insulin sensitivity in T2DM patients (161–163). However, studies demonstrating efficacy of TZDs in cancer chemoprevention are difficult to perform because they should ideally be prospective and involving large cohorts of T2DM patients followed up for several years. Moreover, many confounders may affect such studies because T2DM patients are heterogeneous and often subjected to multiple therapies. Therefore, it is not surprising that only few retrospective studies are available and that their results are largely inconclusive.

A recent retrospective study has assessed the influence of TZDs on the risk of lung, prostate, and colon cancers in patients with diabetes. The study population was derived from the Veterans Integrated services Network 16 data warehouse (164). A total of 87,678 male patients met the study inclusion criteria, and 11,289 were treated with TZDs for a median duration of 1 year. After adjusting for covariates (age, ethnicity, body mass index, HbA1c, use of insulin, or other agents), the use of RGZ or PIO was found to significantly (*P* = 0.0033) reduce the incidence of lung cancer by 33%, as compared with TZDs non-users. A trend for a reduced risk for prostate and colorectal cancer was also observed when the population was analyzed as a whole. However, when results were subjected to subgroup analysis by ethnicity, prostate cancer resulted significantly increased by 15% in white patients while colorectal cancer was significantly reduced in African-Americans. These differences may be explained by possible differences in the metabolism of TZDs between white and African-American populations as well as to the low statistical power of the study (164). Although this study included a large number of patients and had several strengths, it showed several limitations. First, this is a retrospective study, thus some relevant information, such as smoking history and duration of TZD exposure were missing, and other information, such as ethnicity, were not available for all patients.

A different conclusion was reached by a similar retrospective study performed in 1,003 subjects enrolled in the Vermont Diabetes Information System (165). In this community-based diabetic population, the use of TZDs was found significantly and positively associated with the diagnosis of cancer (OR = 1.59, *P* = 0.04). This association was stronger in women (OR = 2.07, *P* = 0.01) and in RGZ users than in PIO users. This study has also several limitations, including the relatively small number of patients, the uncertain duration of TZDs treatment, the lack of diagnosis and tumor stage confirmation, and of rigorous controls for genetic and environmental confounders (165).

A recent meta-analysis has been performed in diabetic patients using RGZ with a focus on cancer risk. This meta-analysis includes all trials that can be retrieved from the GSK (GlaxoSmithKline) web site or from Medline with results published up to February 2008. The authors analyzed 80 trials involving 16,322 RGZ users and 12,522 patients using a different antidiabetic treatment. They found no evidence that RGZ was either positively or negatively associated with cancer risk (OR = 0.91, *P* = 0.44) (166). Case–control studies for specific cancers have also reached discrepant results. In a hospital-based case–control study, carried out in 420 patients (140 diabetics) with HCC and 1,104 controls (115 diabetics), diabetes was found a significant risk factor for HCC, and the treatment with TZDs or biguanides was associated with 70% HCC risk reduction as compared to other treatments. In a similar case–control study involving 973 patients (259 diabetics) with pancreatic adenocarcinoma and 863 controls (109 diabetics), it was found that metformin users, but not TZDs users, were protected from pancreatic cancer as compared to diabetic patients subjected to other treatments (167). Moreover, the risk of breast, colon, and prostate cancers in TZDs users was evaluated in three-nested case–control studies based on 26,971 diabetic patients included in the US Integrated Healthcare Information Services database (168). Data revealed no association between the risk of breast, colon, and prostate cancers and the use of TZDs. The latter study is in agreement with a case–control study performed in 195 diabetic patients and 195 controls, taking into account cancer risk in relation to the antidiabetic treatment used. Also in this study, no association was found between cancer risk and TZD use while a significantly reduced cancer risk was observed in metformin users (166). Yet, a large randomized prospective clinical trial focused on secondary cardiovascular disease prevention in T2DM by PIO (169) has shown that the use of PIO was not associated with significant changes in the incidence of various cancers. The clinical significance of the small increase in bladder cancer (14 vs. 5) and the small decrease in breast cancer (3 vs. 11) remains uncertain. Regarding bladder cancer risk, in another study including a cohort of 193,099 patients with diabetes, the short-term use of PIO was not associated with an increase incidence of bladder cancer, but the use for more than 2 years was weakly associated with increased risk (170). To date, a retrospective cohort study to evaluate the bladder cancer rate in male T2DM subjects aged more than 50 years who are on PIO (7.5–30 mg) therapy for 1 year or more as compared to never users of PIO (http://ClinicalTrials.gov Identifier: NCT01935466, PROBE-PIO) is still ongoing. Similarly, another randomized, double-blind placebo controlled clinical trial evaluating the use of PIO in the chemoprevention of lung cancer (NCT00780234) is also ongoing. In this study, non-diabetic subjects at risk for lung cancer (based on smoking history, lung function testing, and atypical cells in a sputum sample) are randomized to receive either placebo or PIO (30 mg/day). The primary outcome is endobronchial histology and evaluation of cancer progression. Secondary endpoints will include the activation of PPAR-γ dependent signaling pathways.

Taken together, these studies do not provide a definite answer to the question whether the use of TDZs has any association with cancer risk in diabetic population. However, they suggest that, overall, their use is neutral. These results are at odd with those obtained with metformin, which shares with TZDs’ insulin-sensitizing activity, suggesting that the observed effects of these drugs on cancer risk are only partially dependent on their insulin sensitizing effect. It is also possible, that genetic factors, cancer histotype and/or molecular alterations could significantly modulate the clinical effects of TDZs.

## PPAR-α Agonists as Antitumor Drugs: Cross Talk with the IGF System

Peroxisome proliferator activated receptor-α is the first identified PPAR, and it has been found widely expressed in several tissues including skeletal muscle, liver, hearth, kidney, and intestine (171, 172). PPAR-α can be activated by endogenous ligands such as fatty acids and derivatives (arachidonic acid, leukotriene B4, and non-esterified fatty acids) or by synthetic compounds such as fenofibrate, clofibrate, bezafibrate, and Wy-14,63.

Activated PPAR-α forms heterodimer with RXRα (PPARs/RXRα) and binds to the consensus sequence in the promoter region of target genes (173). As PPAR-α agonists modulate the expression of genes regulating glucose and fatty acid metabolism, they have been widely sought after as therapeutic agents for several metabolic disorders such as obesity, T2DM, hyperlipidemia, and cardiovascular diseases (174). However, PPAR-α has also a critical role in the regulation of cell proliferation, survival, motility, and metabolism in several cancer cells (175, 176). Activated PPAR-α may decrease or induce tumor progression depending on the specific tissue or the PPAR-α ligand. While few *in vivo* studies regarding the effects of PPAR-α agonists in cancer are currently available, several *in vitro* studies indicate that PPAR-α has a remarkable antineoplastic potential. For instance, fenofibrate regulates colon inflammation and proliferation by inhibiting the production of pro-inflammatory cytokines such as IL-17, IFN-g, CXCL10, CCL2, and CCL20 (177). In breast and ovarian cancer cells, clofibrate induces HIF-1α degradation leading to decreased tumor-associated VEGF gene expression (178). Yet, in non-small cell lung cancer, activated PPAR-α increases p53 expression and inhibits PDK1 and NFKB/p65 dependent signaling, leading to inhibition of cell growth (179).

Notably, similar to PPAR-γ, PPAR-α may be involved in tumorigenesis by cross talking with the IGF system at different levels. For instance, in brain tumors, fenofibrate exerts anticancer effect by inhibiting IGF-I-mediated signaling and biological responses (180, 181). In medulloblastoma cell lines, fenofibrate strongly inhibited IGF-I-mediated activation of IRS-1, AKT, ERK, and GSK3b phosphorylation (181) as well as IGF-I-mediated cell clonogenic growth, migration, and colony formation in soft agar. Accordingly, the combined treatment with fenofibrate and the IGF-IR inhibitor (NVP-AEW541) resulted in a complete suppression of growth responses to IGF-I, cell cycle arrest, and apoptosis. Similarly, fenofibrate inhibited IGF-I and serum-induced motility of glioma cells and induced ROS accumulation, loss of mitochondrial membrane potential, and reduction in adenosine 5′-triphosphate (ATP) production (180).

In mice bearing 4-nitroquinoline 1-oxide-induced lung hyperplasia, adenoma, and adenocarcinoma (182), fenofibrate downregulated the IGF axis by significantly reducing insulin and IGF-1 serum levels and the immunohistochemical expression of IGF-IR, pAkt, and pERK1/2, indicating a potential as chemoprevention agent. Fenofibrate-mediated inhibition of constitutively activated PI3K/AKT signaling has been reported in melanoma cell lines where this drug decreased both cell invasiveness and clonogenic cell growth (183).

So far, the molecular mechanisms involved in IGF-IR signaling inhibition by fenofibrate are still under investigation and may be at least partially PPAR-α independent, involving changes in the fluidity of plasma membrane, which, in turn, affect ligand-mediated IGF-IR partitioning and consequent initiation of growth-promoting downstream signaling. Indeed, fenofibrate may affect the activities of integral membrane proteins, leading to plasma membrane rigidity and alteration in the activity of membrane-spanning proteins such as IGF-IR (184).

In addition to the attenuation of IGF-IR signaling responses, another mechanism through which fenofibrate exerts antitumor role is the modulation of the energy metabolism of cancer cells, which strongly depend on glycolysis (185). As fenofibrate can switch energy metabolism from glucose to fatty acid oxidation and ketogenesis, as a main source of energy (186, 187), it can favor aberrant mitochondrial oxidative phosphorylation leading to ROS accumulation, oxidative damage, and reduction in ATP production with severe cell energy depletion. As seen in glial neoplasms (180), all of these events may impair growth and survival of cancer cells with defective mitochondrial function. The ability of fenofibrate to force mitochondrial oxidative respiration in tumor cells, without systemic toxicity, suggests that this drug may have clinical benefits in preventing tumorigenesis.

## Conclusion

It is now widely accepted that systemic diseases, such as obesity, T2DM, and metabolic syndrome, are not only cardiovascular risk factors but also cancer risk factors. Insulin resistance and hyperinsulinemia, common features of all these metabolic disorders, contribute to the deregulation of the insulin/IGF axis that plays an important role in cancer progression as well as in CSCs. In light of these considerations, the pleiotropic effects of PPAR-γ agonists may have potential applications in cancer prevention and therapy. Indeed, PPAR-γ activation, by TZD or other agonists, improves insulin resistance and reduces circulating levels of insulin and free IGF-I. Furthermore, PPAR-γ agonists downregulate key pathways of the insulin/IGF axis, such as PI3K/mTOR, MAPK, and GSK3β/Wnt/β-catenin cascades, which regulate cancer cell growth, proliferation, cell reprogramming, and differentiation. Thus, PPAR-γ agonists have a great potential to be used as antineoplastic agents in combination therapies with a variety of other compounds. Although extensive *in vitro* evidence supports the concept of pleiotropic antineoplastic actions of PPAR-γ agonists, unfortunately, the available clinical trials have reached conflicting results, possibly because they have not selected tumors characterized by overactivation of the insulin/IGF-I axis or occurring in hyperinsulinemic patients. Hopefully, future clinical trials with PPAR-γ agonists will include patients with these characteristics. Similarly, studies aiming to show a role of PPAR-γ agonists in cancer prevention should ideally be prospective, randomized, and should involve large cohorts of patients followed up for several years.

Besides PPAR-γ, agonists, also PPAR-α agonists, such as fenofibrate can also inhibit IR/IGF-IR signaling responses and exert complex antineoplastic actions.

In conclusion, the use of PPAR-γ agonists alone or in combination with other agents in cancer prevention and therapy remains promising but still awaits future developments.

## Author Contributions

The authors listed below gave the following contributions. VV, MN, SG, MB, AB, and RM: substantial contributions to the conception and design of the paper; final approval of the version to be published; and agreement to be accountable for all aspects of the work in ensuring that questions related to the accuracy or integrity of any part of the work are appropriately investigated and resolved; VV, MN, SG, and MB: drafting the work; VV, RM, and AB: revising it critically for important intellectual content.

## Conflict of Interest Statement

The authors declare that the research was conducted in the absence of any commercial or financial relationships that could be construed as a potential conflict of interest.

## Abbreviations

PPARs, peroxisome proliferator activated receptors; TZDs, thiazolidinediones; IGF, insulin-like growth factor; T2DM, type 2 diabetes mellitus; IR-A, insulin receptor isoform A; BMI, body mass index; FFA, free fatty acids; IGF-BP1, IGF-binding protein 1; IGF-BP2, IGF-binding protein 2; NK, natural killer; TNF-α, tumor necrosis factor α; IL-6, interleukin-6; ER, endoplasmic reticulum; CAAs, cancer-associated adipocytes; CAFs, cancer-associated fibroblasts; TAMs, tumor-associated macrophages; UPR, unfolded protein response; ATP, adenosine 5′-triphosphate; GPR78, glucose-regulated protein 78; ULK1, unc-51 like autophagy activating kinase 1; RXR, retinoid X receptor; PPRE, PPAR-responsive elements; RGZ, rosiglitazone; PIO, pioglitazone; CSCs, cancer stem cells; TCF/LEF, T cell factor/lymphoid enhancer-binding factor; TKIs, tyrosine-kinase inhibitors; CML, chronic myelogenous leukemia; HCC, hepatocellular carcinoma; DBD, DNA-binding domain; CDKs, cyclin-dependent kinases; RhoB, rho-related GTP-binding protein; Rb protein, retinoblastoma-associated protein; Bcl-2, B-cell lymphoma 2; Bcl-xL, B-cell lymphoma-extra large; Cox-2, cyclooxygenase-2; VEGF, vascular endothelial growth factor; EMT, epithelial to mesenchymal transition; TERT, telomerase reverse transcriptase; ENAH, enabled homolog; ETS, E26-transformation specific family; STAT, signal transducer and activator of transcription; AP1, activator protein-1.
